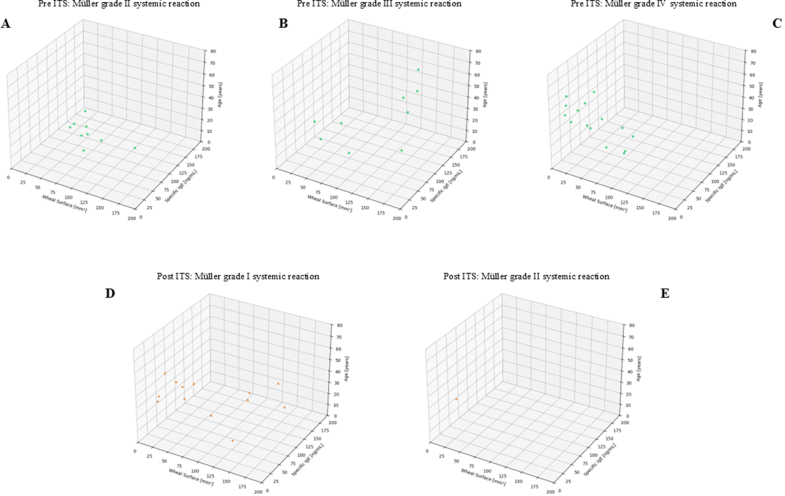# Response to venom immunotherapy: Exploratory retrospective machine learning clustering analysis

**DOI:** 10.1016/j.jacig.2026.100651

**Published:** 2026-01-29

**Authors:** Stefano Palazzo, Marcello Albanesi, Mattia Cristallo, Francesco Pugliese, Nada Chaoul, Giulia Caffarella, Alessandro Cinquantasei, Cataldo Piarulli, Marco Cinquantasei, Marco Aprigliano, Roberto Caldelli, Claudio Loconsole, Silvio Tafuri, Eustachio Nettis, Attilio Di Girolamo

**Affiliations:** a Department of Engineering and Sciences, Universitas Mercatorum, Rome, Italy; b “M. Albanesi” Allergy and Immunology Unit, Bari, Italy; c The Allergist, Bari, Italy; d Allergolys, Paris, France; e University of Bari, Bari, Italy; f Allergy and Immunology Division, Policlinic of Bari, Bari, Italy; g Interdisciplinary Department of Medicine, Aldo Moro University of Bari, Bari, Italy; h Department of Electrical and Information Engineering, Polytechnic of Bari, Bari, Italy; i CNIT—National Interuniversity Consortium for Telecommunications, Florence, Italy; j Institute of Mechanical Intelligence, Scuola Superiore Sant’Anna, Pisa, Italy; k Department of Biomedical Science and Human Oncology, Aldo Moro University of Bari, Bari, Italy

**Keywords:** Hymenoptera, specific IgE, wheal surface area, skin test, immunotherapy, semiautomated method, *k*-means clustering, machine learning, intradermal reactions, artificial intelligence

## Abstract

**Background:**

Hymenoptera venom immunotherapy is an established treatment for severe allergic reactions, aiming to modulate the immune response and reduce allergen sensitivity. However, traditional methods such as skin tests and specific IgE quantification often lack precision and fail to capture the multidimensional nature of clinical data.

**Objective:**

We investigated the relationships between wheal surface area, specific IgE levels, and patient age in allergic reactions to Hymenoptera venom, assessing immunotherapy effects before and after treatment.

**Methods:**

Data were retrospectively collected from 30 patients who underwent intradermal testing before and after immunotherapy. Wheal surface areas were measured using the semiautomated method (SAM), and specific IgE levels via immunoassays. *K*-means clustering, an unsupervised machine learning technique, was applied to identify patient subgroups on the basis of an integrated analysis of the 3 variables. Data normalization ensured comparability across different units.

**Results:**

A positive correlation between wheal surface area and specific IgE was observed before and after treatment, both showing reductions after immunotherapy. Age showed no significant influence. Clustering revealed two consistent profiles: response and partial response. The Müller scale confirmed clinical improvement with reduced reaction severity.

**Conclusion:**

Immunotherapy reduces allergic response, as shown by decreased wheal size and IgE level. The integration of SAM and machine learning enables robust analysis of clinical data, supporting personalized allergy management.

## Graphical abstract

**Figure:**
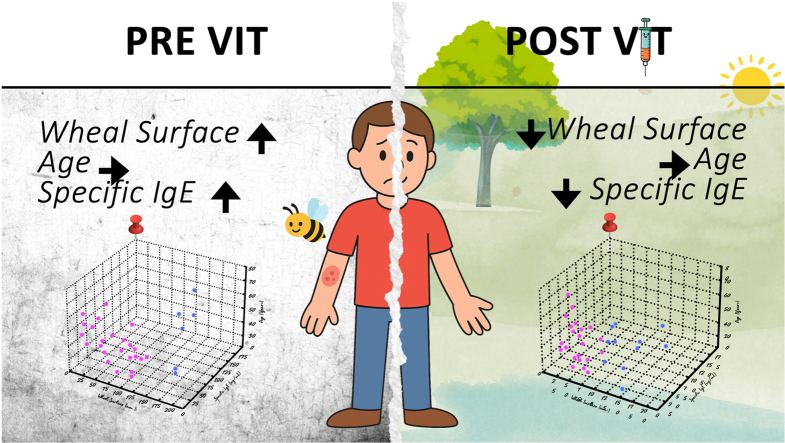

Patients undergoing specific immunotherapy for Hymenoptera venom exhibit a reduction in levels of allergen-specific IgE, indicating a positive change in the patient’s immune response.^1^^,^^2^ This treatment, often used to prevent severe allergic reactions to insect stings such as bees, wasps, and hornets, typically requires a long-term commitment, usually lasting 3 to 5 years. During this period, it is crucial to monitor the longitudinal variations of key variables, including specific IgE levels, to evaluate the efficacy and progress of the therapy.^3^^,^^4^

The scientific literature indicates that the Müller scale is a reliable indicator of the severity of after-sting reactions to insect venom. This scale, widely accepted in the medical community, allows the classification of allergic reactions on a scale from 1 to 4. A score of 1 represents a clinically insignificant reaction, such as slight irritation or redness, while a score of 4 indicates a highly significant reaction, which can include severe systemic symptoms like breathing difficulties, extensive swelling, and anaphylaxis.^5^, ^6^, ^7^

Interestingly, scores on the Müller scale tend to decrease after a complete cycle of immunotherapy. This means that patients who initially have high scores indicating severe reactions can experience a reduction in the severity of their reactions to insect stings as the therapy progresses. In many cases, a significant reduction in scores can be observed even during the course of the treatment, before its completion. This progressive improvement is indicative of the effectiveness of immunotherapy in modulating the immune response and reducing sensitivity to specific Hymenoptera venom allergens.^2^^,^^8^

Specific immunotherapy for Hymenoptera venom represents a crucial therapeutic strategy for patients with severe allergies to insect stings, greatly contributing to the reduction of allergic reactions over time. Periodic assessment using the Müller scale, along with monitoring specific IgE levels, allows for tracking the progress of the treatment and making any necessary adjustments to optimize therapeutic outcomes.^2^^,^^4^^,^^5^

There is currently no standardized international method for quantifying skin tests and intradermal reactions to Hymenoptera venom. Quantifying a wheal in these contexts is essential. For example, in the study of Albanesi et al,^9^ the wheal after intradermal reaction was quantified by measuring the maximum width of the skin reaction to the intradermal test. Further, there are at present few technologically advanced methods for quantifying wheals. One of these is the semiautomated method (SAM) developed by Palazzo et al,^10^ which is fast, precise, and applicable to any type of wheal, both for skin prick tests and intradermal reactions. SAM is used to analyze images from skin tests. The operator manually selects the major and minor diameters of the wheal, and the system provides a numerical quantification of the wheal’s surface area as output. This process indirectly converts variations in color and shape into precise quantitative data, representing a significant improvement over traditional subjective manual evaluations. This innovative method offers greater accuracy in quantifying the wheals resulting from skin tests and intradermal reactions, thus providing a reliable basis for data analysis.

The clinical relevance of SAM lies in its ability to provide more accurate and reproducible data, which can positively influence therapeutic decisions and the management of allergic patients. Precise quantification of the wheal size allows for a detailed evaluation of the immune response, which is crucial for personalizing immunotherapy treatments and improving clinical outcomes.^11^^,^^12^

SAM is of particular importance because it allows for precise quantification of wheal size and provides an opportunity to subsequently examine how these variables correlate with specific IgE levels (relative to the same allergen) and the age of the analyzed patients. In our clinical practice, in addition to wheal quantification, patients who have completed immunotherapy for Hymenoptera venom may undergo ImmunoCAP for the quantification of specific IgE related to the allergen for which the immunotherapy was performed. In this study, we performed this test before and after immunotherapy for exploratory characterization only. Typically, after immunotherapy, a decrease in specific IgE relative to total IgE may be observed, although current guidelines do not recommend using IgE levels to assess individual efficacy.^1^^,^^2^^,^^8^

Because venom immunotherapy treatment may span over 5 years, patient aging can play a significant role in the context of the biological response to the treatment. The efficacy and tolerance of immunotherapy can vary according to patient age, so therapeutic decisions must be adjusted accordingly. For instance, in children, immunotherapy is generally considered when the allergic reaction is systemic and severe, so risks and benefits must be carefully evaluated. In adults and the elderly, the immune response and potential adverse effects of immunotherapy may differ.^13^, ^14^, ^15^

It is known that the relationship between specific IgE levels, wheal size in skin tests, and patient age is a complex but crucial aspect in diagnosing and managing allergies. Specifically, specific IgE antibodies are produced by the body in response to specific allergens. An increase in these antibodies indicates allergic sensitization, but their levels do not always directly reflect the severity of clinical symptoms.^16^^,^^17^ Wheal size, which is measured in skin tests such as the skin prick test, provides a visible indication of the patient’s allergic reaction.^18^ In particular, a wheal size of >3 mm compared to negative control suggests significant sensitization to the tested allergen.^19^^,^^20^

Patient age is another critical factor to consider.^21^ In children, allergies are often more pronounced, with potentially higher levels of IgE, which may decrease over time or as the immune system develops.^22^^,^^23^ In adult populations, age-related immune changes (immunosenescence) may also modulate allergic sensitization and test reactivity, with several studies reporting lower total and/or specific IgE and reduced sensitization rates with advancing age, while evidence on wheal size is heterogeneous and context dependent. In light of this mixed literature across the life-span, we therefore included age *a priori* as an exploratory covariate rather than as a primary causal hypothesis in our adult cohort.^24^, ^25^, ^26^, ^27^

Therefore, an integrated understanding of these 3 factors—specific IgE, wheal size, and patient age—is essential for accurate diagnosis and effective management of allergies. The combination of these 3 parameters allows for personalized treatment, improving the therapeutic approach and the patient’s quality of life.^28^^,^^29^

The current literature does not provide comprehensive information on the relationships between age, specific IgE levels, and wheal size in patients allergic to Hymenoptera venom, particularly concerning immunotherapy.^11^^,^^30^ Although several studies describe reductions in venom-specific IgE levels and/or skin test wheal size during venom immunotherapy, these biomarkers are not reliable indicators of clinical protection, and current guidelines from the European Academy of Allergy and Clinical Immunology, the American Academy of Allergy, Asthma & Immunology, and the British Society for Allergy & Clinical Immunology do not recommend monitoring them to assess individual efficacy or relapse risk. In venom allergy, venom-specific IgE results and skin test wheal size do not consistently correlate with reaction severity.

What remains insufficiently explored, however, is a purely exploratory, multivariate characterization of how age, wheal size, and specific IgE covary before and after venom immunotherapy—without inferring clinical efficacy—which could inform hypothesis generation for future studies.^14^^,^^31^

Three-dimensional analysis of data collected before and after immunotherapy may provide new insights into treatment effectiveness as well as the interaction between these variables in allergic skin reactions. While some studies have explored specific relationships, such as between specific IgE levels and wheal size, a combined analysis that also includes age is lacking. Understanding how these 3 variables interact and influence therapeutic response is crucial.^9^^,^^32^ One method to study the relationship between age, specific IgE levels, and wheal size in a clinical context is clustering, such as the application of *k*-means clustering, which allows patients to be divided into homogeneous groups.^33^ This method helps identify clusters of patients with similar characteristics, facilitating the analysis of the relationships between variables.^34^ Moreover, to ensure greater data uniformity and minimize potential biases, studies of this kind should be conducted on cohorts of patients from the same geographic area because they share similar environmental and demographic factors.^35^, ^36^, ^37^

Clustering techniques not only enhance the visual understanding of data but also provide a deeper interpretation of the interactions among variables, contributing to a more detailed and targeted analysis of patients’ clinical characteristics.^38^ Unsupervised machine learning (ML), such as clustering, is crucial in biomedical engineering for assessing and optimizing targeted treatments and interventions that are based on the specific characteristics of the local population, offering the added benefit of being computationally efficient.^39^^,^^40^

In this study, we utilized ML techniques, such as nonhierarchical *k*-means clustering, to analyze data and identify hidden patterns. We aimed to analyze the postimmunotherapy treatment progress using a 3-D visualization that correlated with the wheal surface area, specific IgE levels, and patient age. Additionally, we examined the clustering of data after we applied the *k*-means clustering algorithm. This analysis provided important insights into the efficacy of the immune response induced by immunotherapy. This approach allowed for the segmentation of the dataset into homogeneous groups, facilitating the identification of significant correlations between the variables studied. The combination of ML techniques and SAM represents an innovative advancement in allergy research, offering significant potential clinical benefits.

## Methods

### Data collection

The data were provided by the Department of Allergy and Clinical Immunology of the Polyclinic of Bari (Italy) and pertained to patients who were considered candidates for immunotherapy after specialist evaluation for allergic symptoms. Our study had a retrospective observational design that was based on real-world data collected from patients who had already undergone specific immunotherapy according to standard clinical indications. We adopted an observational and exploratory approach; we did not aim for a controlled experimental design.

The recruited patient group consisted of 30 subjects, with data collected in a manner that ensured complete anonymity. The mean ± standard deviation age at the beginning of recruitment (before immunotherapy) was 43.85 ± 14.23 years, while at the end of recruitment (after immunotherapy) it was 48.85 ± 14.23 years. Among the total patients, 22 received immunotherapy for *Vespula* spp *europea*, 6 for *Polistes dominulus*, 3 for *Apis mellifera*, and 2 for *Vespa crabro*.

Although our study enrolled a total of 30 patients, 3 of them received immunotherapy for two different venoms. Considering the potentially distinct immunologic response induced by each venom, these cases were treated as separate observations, bringing the total number of analyzed data points to 33. Table I lists the variables measured before and after Hymenoptera venom immunotherapy: specific IgE, allergen-induced wheal surface area, histamine wheal, age, Müller grade, and immunotherapy.

**Table I:** Variables measured before and after Hymenoptera venom immunotherapy

Patient no.	Before therapy					After therapy					Immunotherapy
	Specific IgE (kU_A_/L)	SAM allergen wheal surface (mm^2^)	Age (years)	SAM histamine wheal surface (mm^2^)	Müller grade systemic reaction	Specific IgE (kU_A_/L)	SAM allergen wheal surface (mm^2^)	Age (years)	SAM histamine wheal surface (mm^2^)	Müller grade systemic reaction	
1	0.68	25.23	50	71.34	4	1.31	126.27	57	113.82		*Vespula* spp *europea*
2	2.28	105.62	55	80.48	3	0.7	76.45	60	86.87	1	*Vespula* spp *europea*
3	10.6	104.83	24	70.22	3	1.01	79.4	33	116.67		*Vespula* spp *europea*
4	0.16	74.39	77	91.48	4	0.06	54.87	82	77.66		*Vespula* spp *europea*
5	80.6	121.05	35	80.37	3	6.28	36.48	40	67.56	2	*Apis mellifera*
6	16.6	92.66	33	103.94	2	2.57	67.21	39	133.61		*Polistes dominulus*
7	21.1	67.58	33	103.94	2	2.7	84.56	39	133.61		*Vespula* spp *europea*
8	4.5	141.38	45	57.65	2	13.4	119.87	50	33.02		*Vespula* spp *europea*
9	16.6	41.45	31	94.99	4	33.8	132.43	36	107.05	1	*Vespa crabro*
10	21.1	104.14	31	94.99	4	41.4	124.69	36	107.05		*Vespula* spp *europea*
11	4.34	99.18	52	74.46	2	1.25	60.65	57	76.1		*Vespula* spp *europea*
12	2.03	82.98	55	66.91	4	9.59	219.69	60	53.16	1	*Vespula* spp *europea*
13	1.74	59.29	46	111.71	4	1.25	78.13	50	67.97	1	*Vespula* spp *europea*
14	4.68	68.36	34	56.59	3	0.84	71.98	39	70.04		*P dominulus*
15	25.6	87.42	34	77.93	2	2.26	32.89	40	80.56	1	*Vespula* spp *europea*
16	2.96	117.63	33	71	4	5.29	148.32	39	36.63		*Vespula* spp *europea*
17	37.5	153.73	61	69.22	3	5.51	130.46	66	81.37		*Vespula* spp *europea*
18	3.47	118.27	66	91.98	2	3.36	57.09	72	67.28		*Vespula* spp *europea*
19	5.06	58.49	47	65.41	3	8.38	101.59	52	114.45		*Vespula* spp *europea*
20	14.9	181.49	36	92.7	3	3.1	117.18	41	48.7	1	*A mellifera*
21	33.7	165.75	53	57.15	3	9	81.78	58	77.02	1	*A mellifera*
22	8.04	108.38	26	123.69	4	0.29	59.95	32	69.78		*P dominulus*
23	0.8	58.58	65	84.42	4	0	143.73	71	56.76		*V crabro*
24	33.3	155.05	21	60.68	2	2.29	151.75	26	84.2	1	*P dominulus*
25	20.7	96.56	32	128.53	2	16.8	90.26	37	59.39		*Vespula* spp *europea*
26	4.38	113.77	32	128.53	2	2.97	63.21	37	59.39		*P dominulus*
27	11.7	74.26	23	99.41	4	5.38	80.63	28	93.98		*Vespula* spp *europea*
28	83.4	118.29	52	75.68	3	34.36	178.85	57	99.45	1	*Vespula* spp *europea*
29	5.1	110.7	50	95.78	4	0	74.71	56	94.84		*Vespula* spp *europea*
30	0.01	47.62	58	68.59	4	0.05	67.47	63	91.59	1	*Vespula* spp *europea*
31	6.81	18.56	61	137.56	4	2.01	46.72	66	127.41	1	*Vespula* spp *europea*
32	4.54	21.1	55	63.98	4	0.6	58.5	60	80.67		*P dominulus*
33	5.92	26.39	41	78.51	4	0.74	37.58	46	95.41	1	*Vespula* spp *europea*

Variables included: specific IgE (kU_A_/L), allergen-induced wheal surface area (mm^2^, SAM), age (years), histamine wheal (mm^2^, SAM), and Müller grade. *Immunotherapy* indicates type of Hymenoptera venom treatment and is constant between before-and-after measures.

The relatively small number of patients included in this study is primarily due to the highly specific inclusion criteria and the clinical context in which the research was conducted. Patients were selected on the basis of a confirmed diagnosis of Hymenoptera venom allergy and their eligibility for specific immunotherapy after a specialist allergy evaluation. The analysis was conducted exclusively on patients who experienced a clinically positive response to treatment. Therefore, the results should be interpreted as preliminary but also as representative of potentially broader trends, providing a solid foundation for future studies on larger and multicenter cohorts.

We aimed to examine the relationships between wheal size, specific IgE levels, and the age of patients. These data pertain to the pre- and postimmunotherapy results for intradermal reactions related to Hymenoptera venom. Each patient was represented by a combination of clinical variables, including age (years), specific IgE levels (kU_A_/L), and wheal surface area (mm^2^). In addition, the severity of the systemic reaction to Hymenoptera venom was graded using the Müller scale and clinician documentation at the time of evaluation. For this study, the grade was abstracted verbatim from the medical record; we did not perform retrospective regrading. This variable was used for clinical characterization and was not included in the clustering analyses.

In particular, we used two techniques to quantify wheal surfaces: SAM, an automated system developed by Palazzo et al,^10^ and the traditional technique, which involves measuring the mean diameter of the wheal after a skin prick test using graph paper. The wheal’s surface area is then calculated from the diameter.

The entire data analysis for this project was conducted by SAM, an innovative technique that combines the efficiency of automation with the precision of manual analysis.^10^ For the present retrospective observational analysis, SAM was applied *a posteriori* to preexisting archived intradermal test images obtained during routine care (both before and after immunotherapy); no procedures beyond standard clinical practice were performed. No sting challenge was performed; provoked-sting outcomes are not available in our clinical setting because such procedures are not authorized in Italy, and in many European contexts, they are limited to specific research protocols.

The study received approval from the relevant ethics committee (approval 1684/CEL).

### Data preparation

The units of measure we used were years for age, square millimeters for wheal surface area, and nanograms per milliliter for specific IgE, with the latter converted from its classical expression (kU_A_/L, typical of ImmunoCAP results). To convert specific IgE from kU_A_/L to ng/mL, it is necessary to consider that 1 kU_A_/L of IgE is equivalent to 2.4 ng/mL, based on the average molecular weight of the IgE antibody. Therefore, the IgE value in kU_A_/L must be multiplied by 2.4 to obtain the corresponding value in ng/mL. A preliminary data analysis was conducted using the original values, which were displayed in the selected units of measure. We subsequently applied *k*-means clustering to this dataset, specifying a division into two clusters. This process was carried out for both the pre- and postimmunotherapy patient data.

### Data normalization

Because the units of measure of the 3 variables were not the same, some values were numerically larger than others. It was therefore essential to prevent features with significantly different scales from disproportionately influencing the clustering process. To achieve this, StandardScaler (from ‘scikit-learn,’ an open-source Python library specialized in ML) was applied, standardizing the data by setting the mean to 0 and the variance to 1.^41^ Afterward, the data were normalized to ensure that all 3 variables (wheal surface area, specific IgE, and age) had the same weight. Data normalization was implemented by using statistical techniques that transformed values into proportional scales, thus allowing for a fair and accurate comparison between the various parameters, regardless of their original units of measure.

After normalizing the data, a weighting adjustment was applied: Wheal surface area was assigned a weight increased by ⅔, specific IgE levels were weighted with an increase of ⅓, and age retained its original weight. Subsequently, *k*-means clustering was performed on this normalized dataset. As in the previous analysis, the number of clusters was set to 2, and the results were visualized for both pre- and postimmunotherapy data.

### Data analysis and methodologic approach

The advanced ML technique of nonhierarchical clustering using the *k*-means method was used to identify hidden patterns in the data. We selected *k*-means clustering because of its efficiency, suitability for continuous clinical variables, and ability to generate easily interpretable patient groupings, making it appropriate for exploratory stratification in a real-world clinical setting. This unsupervised approach was adopted because no reliable clinical labels were available for *a priori* classification; clustering allowed for the exploration of latent patterns within the data and enabled objective stratification that was solely based on the analyzed variables. Indeed, the use of the nonhierarchical *k*-means clustering technique allowed the dataset to be divided into homogeneous groups on the basis of the similarity of observed characteristics, facilitating the identification of intrinsic patterns and correlations in the allergy-related data. The goal was to group the observations into clusters to maximize similarity within each cluster and minimize similarity between different clusters. The *k*-means clustering algorithm helped us identify patterns and hidden groups within the data, revealing similarities and differences that may not be immediately apparent.

The *k*-means algorithm works by minimizing intracluster variance, iteratively adjusting the positions of the centroids and reassigning data points to the nearest centroid until convergence is reached. This process ensures that each data point is grouped with others that have similar characteristics, resulting in homogeneous clusters.^42^^,^^43^ In our specific case, the optimal number of clusters was set to two. This decision was based on a balance between ensuring interpretability for clinicians and minimizing computational complexity. The decision to set the number of clusters to two was deliberately made to reflect the simplest and most clinically interpretable scenario: the division of patients into two main groups, one with a more severe allergic response (potentially severe) and the other with a milder response (less severe). This binary approach represented a simplified yet effective clinical model that enabled initial stratification of patients according to the severity of their immunologic response.

Although various techniques are available for automatically determining the optimal number of clusters, the primary objective of this study was not to identify the statistically optimal number of clusters but rather to evaluate the separation and evolution of two clinically relevant categories before and after immunotherapy treatment.

### Data analysis tools

The programming language used for the implementation of all analyses and procedures was Python, while data statistical analysis and figure generation were performed by MATLAB.

### Linguistic support and translation

ChatGPT 4.0 (OpenAI) was used solely to support the translation of specific phrases and technical terms from Italian to English. No content was generated by artificial intelligence (AI); its role was strictly limited to linguistic assistance. The scientific literature acknowledges ChatGPT as a valuable tool for nonnative speakers to improve academic texts, thereby enhancing international scientific communication.^44^, ^45^, ^46^, ^47^, ^48^

AI-generated outputs were reviewed and validated by a domain expert to ensure accuracy. As noted by Palazzo et al,^10^ the optimal approach combines human expertise with AI capabilities.

## Results

### Clinical efficacy of venom immunotherapy

**Fig 1:**
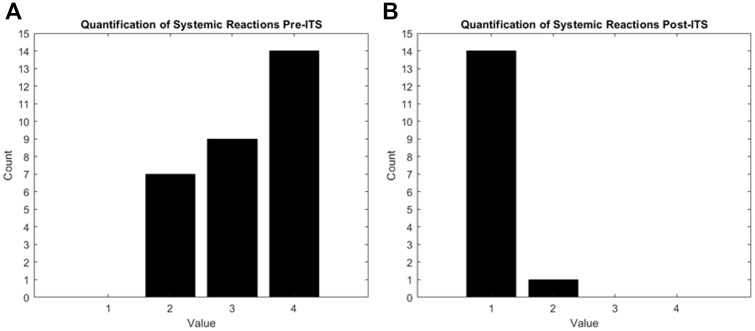
Quantification of systemic reactions before **(A)** and after **(B)** Hymenoptera venom immunotherapy according to Müller scale.

The Müller reactions objectively demonstrated the effectiveness of immunotherapy. Fig 1, *A,* corresponding to the preimmunotherapy phase, shows an increase in Müller scale values, whereas Fig 1, *B,* corresponding to the postimmunotherapy phase, highlights a reduction in these values. Fig E1 in the Online Repository available at www.jaci-global.org presents a graphical representation of the data stratified according to the Müller grades observed in patients before and after immunotherapy. In the pretreatment phase, only grades II, III, and IV were identified, whereas in the posttreatment phase, only grades I and II were observed; consequently, only these grades were included in the respective plots. For each grade considered, individual patient-specific observations were plotted within a 3-D space defined by our selected variables: wheal surface area, specific IgE concentration, and age.

**Fig 2:**
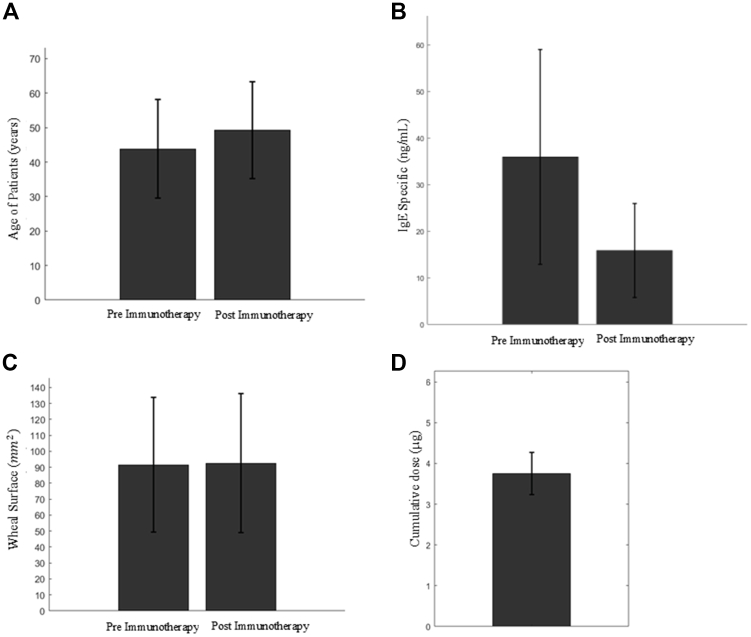
Histogram showing mean and standard deviation of patients’ age before *(left)* and after *(right)* immunotherapy **(A)**. Histogram showing mean and standard deviation of specific IgE concentrations (ng/mL) in patients before *(left)* and after *(right)* immunotherapy **(B)**. Histogram showing mean and standard deviation of wheal areas (mm^2^) induced by allergen in patients before *(left)* and after *(right)* immunotherapy **(C)**. Histogram showing mean and standard deviation of total allergen dose administered during immunotherapy period (μg), calculated according to number of treatment years and fixed administration schedule (0.1 mg every 8 weeks) **(D)**.

Additional data analysis was conducted on the 33 patient observations. In Fig 2, *A,* the mean age of patients before and after immunotherapy is shown, with values of 43.85 ± 14.23 years and 48.85 ± 14.23 years, respectively, considering that immunotherapy was administered at different times for each patient. By paired Student *t* test, the *P* value was extremely low (*t* = −35.87; *P* ≈ 2.05 × 10^−27^), indicating a highly statistically significant difference.

The mean and standard deviation values of specific IgE levels were also calculated before and after immunotherapy, resulting in 35.99 ± 23.08 ng/mL and 15.89 ± 10.08 ng/mL, respectively (Fig 2, *B*). A paired Student *t* test yielded *P* = .0127 (*t* = 2.641), a statistically significant difference. The same approach was applied to the wheal surface area, which showed values of 91.52 ± 42.25 mm^2^ for the preimmunotherapy phase and 92.59 ± 43.68 mm^2^ for the postimmunotherapy phase (Fig 2, *C*). A paired Student *t* test showed no statistically significant difference between wheal surface values before and after immunotherapy, at *P* > .05 (*t* = −0.115; *P* = .909).

Fig 2, *D,* shows the total amount of allergen administered to each patient throughout the immunotherapy treatment. Each patient underwent immunotherapy for *n*-years, with an average frequency of one administration every 8 weeks (approximately 7 administrations per year), and each dose contained 0.1 mg of allergen. Therefore, the annual amount administered was 700 μg. By multiplying this value by the number of treatment years, the total cumulative dose of allergen administered was calculated.

### Correlation analysis

#### Linear regression

**Fig 3:**
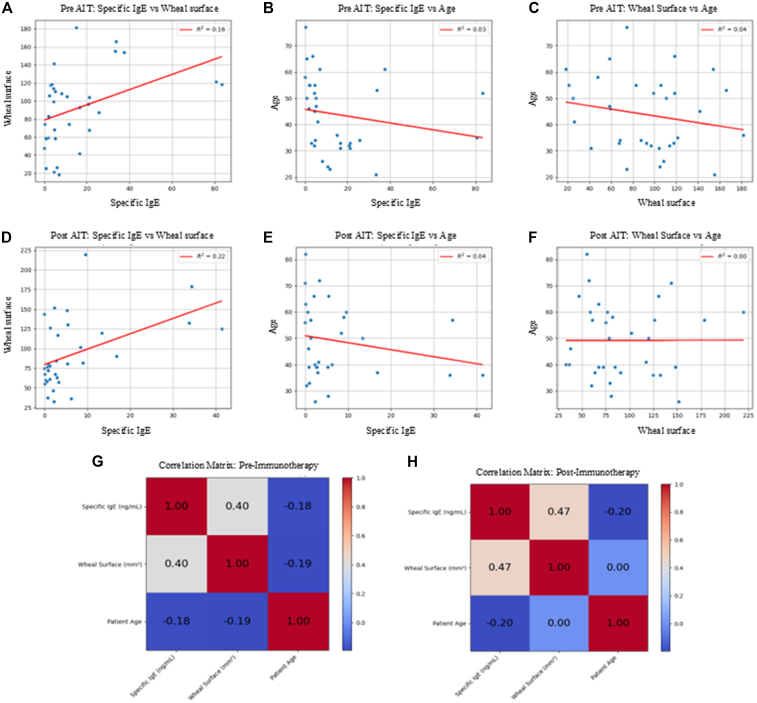
Scatterplot showing relationship between specific IgE levels and wheal surface area in patients before immunotherapy. *Red line* represents linear regression line, with *R*^2^ indicating strength of relationship between 2 variables **(A)**. Scatterplot showing relationship between specific IgE levels and patient age before immunotherapy. *Red line* represents linear regression line, with *R*^2^ indicating strength of relationship between 2 variables **(B)**. Scatterplot showing relationship between wheal surface area and patient age before immunotherapy. *Red line* represents linear regression line, with *R*^2^ indicating strength of relationship between 2 variables **(C)**. Scatterplot showing relationship between specific IgE levels and wheal surface area in patients after immunotherapy. *Red line* represents linear regression line, with *R*^2^ indicating strength of relationship between 2 variables **(D)**. Scatterplot showing relationship between specific IgE levels and patient age after immunotherapy. *Red line* represents linear regression line, with *R*^2^ indicating strength of relationship between 2 variables **(E)**. Scatterplot showing relationship between wheal surface area and patient age after immunotherapy. *Red line* represents linear regression line, with *R*^2^ indicating strength of relationship between 2 variables **(F)**. Correlation matrix, calculated by Pearson correlation coefficient, showing relationship between specific IgE levels (ng/mL), allergen-induced wheal area (mm^2^), and patient age (years) before initiation of immunotherapy **(G)**. Correlation matrix, calculated by Pearson correlation coefficient, showing relationship between specific IgE levels (ng/mL), allergen-induced wheal area (mm^2^), and patient age (years) at end of immunotherapy **(H)**.

In the preimmunotherapy phase, linear regression analysis revealed a weak correlation between specific IgE levels and wheal surface area, with a coefficient of determination of *R*^2^ = 0.16. The correlations between specific IgE and age (*R*^2^ = 0.03), and between wheal surface and age (*R*^2^ = 0.04), were negligible (Fig 3, *A-C*). After treatment, the correlation between specific IgE and wheal surface slightly increased (*R*^2^ = 0.22). The relationships between IgE and age (*R*^2^ = 0.04), and between wheal surface and age (*R*^2^ = 0.00), remained absent or very weak in the posttreatment phase as well (Fig 3, *D-F*).

#### Correlation matrix

The correlation matrix shown in Fig 3, *G,* was computed by the Pearson index to analyze the relationship between specific IgE levels (ng/mL), the wheal surface induced by the allergen (mm^2^), and the patient’s age (years) before the initiation of immunotherapy. Similarly, the correlation matrix presented in Fig 3, *H,* describes the relationships among the same variables at the end of the treatment.

The analysis of Pearson correlation coefficients, computed both in the preimmunotherapy phase and at the end of the treatment, reveals that the correlation between wheal area and specific IgE levels is positive, with values generally high and approaching unity, indicating a moderate to strong direct association. The correlation between patient age and wheal area is close to zero or negative, suggesting the absence of a linear relationship or, at most, a weak inverse association. Similarly, the correlation between patient age and specific IgE levels is predominantly negative, indicating an inverse relationship of variable magnitude.

### 3-D analysis and ML application

A data analysis was conducted aimed at the 3-D representation of our variables of interest to visualize the distribution of observations in the space of the variables considered. In this context, the data relating to the 33 patient observations belonging to our sample were examined, considering 3 different methods of treatment of the variables: original values, standardization, and normalization.

For each of these transformations, the *k*-means clustering algorithm was applied, setting the number of clusters to 2. The analyses were performed separately on the data collected before and after immunotherapy, allowing a visual and quantitative comparison of the patterns that emerged as a function of the therapeutic intervention. The effect of immunotherapy on the allergic response in the analyzed patient cohort was evaluated by applying 3 different approaches to 3-D clustering. We turn to each in turn.

#### Original data

**Fig 4:**
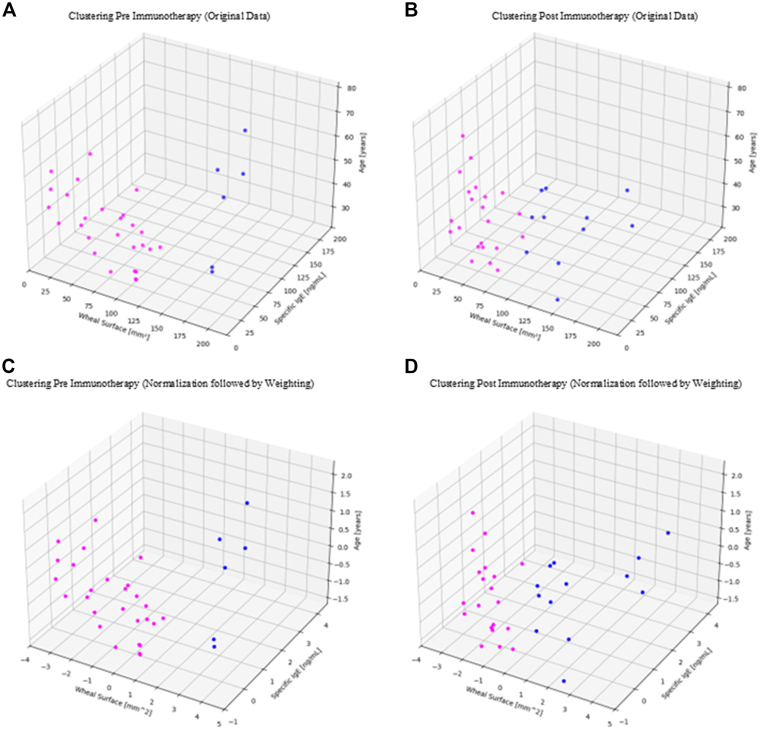
Graph presents 3-D representation illustrating relationship between wheal surface area (mm^2^), specific IgE levels (ng/mL), and patient age (years). Data of 33 patient observations before immunotherapy are displayed, with application of *k*-means clustering algorithm, set to 2 clusters. Clustering was performed on original, nonnormalized data, maintaining units of measure as recorded **(A)**. Graph presents 3-D representation illustrating relationship between wheal surface area (mm^2^), specific IgE levels (ng/mL), and patient age (years). Data of 33 patient observations after completing immunotherapy cycle are displayed, with application of *k*-means clustering algorithm, set to 2 clusters. Clustering was performed on original, nonnormalized data, maintaining units of measure as recorded **(B)**. Graph presents 3-D representation illustrating relationship between wheal surface area (mm^2^), specific IgE levels (ng/mL), and patient age (years). Data of 33 patient observation before immunotherapy are displayed, with application of *k*-means clustering algorithm, set to 2 clusters. Clustering was performed on normalized data, applying different weights to variables according to their clinical relevance: greater weight was assigned to wheal surface area (⅔); intermediate weight to specific IgE levels (⅓); actual weight was maintained for patient age **(C)**. Graph presents 3-D representation illustrating relationship between wheal surface area (mm^2^), specific IgE levels (ng/mL), and patient age (years). Data of 33 patient observations after completing immunotherapy cycle are displayed, with application of *k*-means clustering algorithm, set to 2 clusters. Clustering was performed on normalized data, applying different weights to variables according to their clinical relevance: greater weight was assigned to wheal surface area (⅔); intermediate weight to specific IgE levels (⅓); actual weight was maintained for patient age **(D)**.

Clustering was applied to the untransformed data, maintaining the original units of measure for each variable. The analyses were represented through 3-D graphs of the 3 variables considered: specific IgE (ng/mL), wheal surface caused by the allergen (mm^2^), and patient age (years). The analysis was conducted both on the data collected before the start of immunotherapy (Fig 4, *A*) and on those obtained at the end of treatment (Fig 4, *B*).

#### Normalized data with different weights

Clustering was applied to the standardized and normalized data with differentiated weights between the variables, assigned in accordance with what is reported in the literature. In particular, a greater weight was attributed to the wheal surface (⅔), an intermediate weight to specific IgE (⅓), and the real weight to the patient’s age. The analyses were visualized using 3-D graphs of the 3 variables considered: specific IgE (ng/mL), wheal surface caused by the allergen (mm^2^), and patient’s age (years). The analysis was performed both on the data collected before the start of immunotherapy (Fig 4, *C*) and on data obtained at the end of treatment (Fig 4, *D*).

Indeed, from Fig 4, *D* and *C,* it emerges that before immunotherapy, the clustering analysis revealed a high degree of heterogeneity within the cohort, with a marked asymmetry in the distribution between the two clusters (approximately 81.82% of patients in the larger cluster *[violet]* and 18.18% in the smaller cluster *[blue]*). After treatment, a greater homogeneity is observed in the analyzed population, with a more balanced distribution between the groups (39.39% in one cluster *[blue]* and 60.61% in the other *[violet]*), suggesting a normalizing effect of immunotherapy on allergic response.

The partial overlap of clusters between the pre- and postimmunotherapy phases suggests that despite clinical improvement, variations in the analyzed parameters are distributed gradually and do not result in a clear reclassification of cases.

## Discussion

### Clinical efficacy of venom immunotherapy

We conducted a quantitative analysis of the efficacy of immunotherapy for Hymenoptera venom using the Müller scale to evaluate patients’ reactions. Before immunotherapy, the distribution of patients on the Müller scale was as follows: most cases were classified with a score of 4, followed by cases with scores of 3 and 2, while a single case had a score of 1 (Fig 1, *C*). After immunotherapy, a significant improvement was observed: scores of 3 and 4 completely disappeared, and most cases obtained a score of 1, with only a single case classified with a score of 2 (Fig 1, *D*). The Müller scale was incorporated to ensure comprehensive clinical characterization because it represents a standardized and validated parameter for assessing the severity of systemic reactions in individuals allergic to Hymenoptera venom, aligning with the purpose and objectives of the study.

Fig 2 shows that the patients receiving immunotherapy did not necessarily follow a uniform treatment-time interval; the duration of treatment varied according to individual decisions. After completion of therapy, a significant reduction in specific IgE levels was observed. However, the wheal surface did not show statistically significant variations between pre- and post-treatment assessments, remaining substantially unchanged. This phenomenon can be attributed to the fact that the reduction of skin reactivity to a given allergen is not a predictable or universally expected outcome of immunotherapy.

Overall, our observations were in line with the scientific literature on venom immunotherapy, showing reduced systemic reaction severity and lower venom-specific IgE, but no reliable, uniform change in skin test wheal size.^2^^,^^49^, ^50^, ^51^

### SAM acquisition technique

Two techniques for quantifying wheal surfaces were employed: a traditional technique and SAM. The traditional technique involves measuring the mean diameter of the wheal after a skin prick test using graph paper. Subsequently, the wheal’s surface area was calculated from the diameter.

It has been previously demonstrated by Palazzo et al^10^ that SAM offers greater accuracy in quantifying the wheal surface area. Therefore, *k*-means analysis was conducted using the surface areas quantified through SAM. Consistent with this choice, in the present study, SAM represented an innovative quantification approach—one that has not yet applied in this specific context and that to date has not been used to evaluate intradermal reactions to Hymenoptera venom.

### Correlation analysis

#### Linear regression

In Fig 3, *A-C,* the correlations observed in the preimmunotherapy phase are shown. A weak positive correlation is observed between specific IgE levels and wheal surface: only 16% of the variability in wheal size is explained by IgE levels (*R*^2^ = 0.16). This indicates that although a trend exists, other factors significantly influence cutaneous reactivity.

The correlation between specific IgE and age is negligible: Age accounts for only 3% of the variability in IgE levels (*R*^2^ = 0.03), suggesting that there is no significant association between age and allergic sensitization in the pretreatment phase. In fact, the IgE–age regression line shows an almost flat slope (Fig 3, *B*), confirming that age explains only a minimal portion of IgE variability and exerts a marginal influence on the immunologic response.

Similarly, the relationship between wheal surface and age is very weak, with an *R*^2^ of 0.04. Age has minimal impact on skin reactivity before the start of therapy. As shown in Fig 3, *C,* the wheal–age trend is nearly horizontal, indicating that differences in patient age do not translate into measurable variations in wheal area before treatment.

Fig 3, *D-F,* shows the correlations in the postimmunotherapy phase. The correlation between specific IgE levels and wheal surface slightly increases: 22% of the variability in wheal size is explained by IgE levels (*R*^2^ = 0.22). This increase suggests a greater consistency between residual sensitization and cutaneous response, although the relationship remains weak. In contrast, the correlation between IgE and age remains unchanged compared to the pretreatment phase (*R*^2^ = 0.03); age still does not significantly affect specific IgE levels after therapy. More precisely, the regression line remains almost flat (Fig 3, *E*), indicating that even after immunotherapy, patient age does not exert a measurable effect on IgE levels.

Finally, the correlation between wheal surface and age disappears entirely in the posttreatment phase, with no detectable impact of age on cutaneous reactivity. In the postimmunotherapy setting (Fig 3, *F*), the *R*^2^ approaches zero (*R*^2^ ≈ 0.00), confirming the absence of a linear association between age and wheal size and suggesting that immunotherapy does not introduce any age-related modulation of cutaneous reactivity.

In summary, the picture emerging from our regressions—namely a weak IgE–wheal association, a negligible effect of age on both IgE and wheal, and the absence of a post–venom immunotherapy linear relationship between age and cutaneous reactivity—is fully consistent with the scientific literature, which reports only modest correlations between IgE and wheal size and weak or absent associations of age with venom-specific IgE and with skin test responses in cohorts undergoing Hymenoptera venom immunotherapy.^24^^,^^52^, ^53^, ^54^, ^55^

#### Correlation matrix

Fig 3, *G* and *H,* provides summaries of the correlations between the 3 variables (wheal surface area, specific IgE levels, and age) in the pre- and postimmunotherapy periods. These matrixes offer a detailed understanding of the strength of the associations between each of these variables, providing a quantitative analysis of their interdependence.

The analysis of Pearson coefficients highlights a strong positive association between specific IgE levels and wheal area, suggesting that higher allergen sensitization corresponds to a more extensive cutaneous reaction. Conversely, the correlation matrixes quantitatively confirm this interpretation regarding age. In the preimmunotherapy phase (Fig 3, *G*), the age–IgE and age–wheal cells are close to zero or slightly negative, and the same pattern is observed in the postimmunotherapy phase (Fig 3, *H*). This indicates that patient age exhibits a null or negative correlation with both wheal area and specific IgE levels, suggesting that immunotherapy does not introduce any measurable age-related effect on IgE levels or cutaneous reactivity, and that age plays only a marginal or inverse role in determining the allergic response.

The consistency of these findings between the pre- and postimmunotherapy phases suggests that the treatment does not substantially modify the relationships among these variables, although other potential effects of immunotherapy on the immune response cannot be excluded.

### 3-D analysis and ML application

Biparametric analyses proved to be inadequate in capturing the complexity of the relationships among the variables. However, the correlation matrix revealed greater consistency in the results. Consequently, the adoption of multifactorial analysis tools became necessary. This led us to use ML techniques, which are more effective in integrating and modeling the interactions among the variables under examination.

In this context, the aim of this study was to analyze the relationship among the 3 variables by representing these values in a 3-D graph, to which *k*-means clustering has been applied. Our exploratory retrospective study used the AI technique of unsupervised *k*-means clustering to integrate, in a single 3-D analysis conducted before and after Hymenoptera venom immunotherapy, wheal measurement (assessed by SAM), specific IgE levels, and patient age. The innovative element lies in the simultaneous integration and longitudinal comparison of these 3 domains, which have largely been investigated separately or in a bivariate fashion; indeed, 3-variable clustering provides greater informational power than a 2-variable analysis.^56^, ^57^, ^58^

In the scientific literature, there are studies that jointly consider venom-specific IgE, age, and, in some cases, wheal size, which are variables for which age and IgE sometimes show associations with reaction severity. Wheal size alone is not regarded as a reliable predictor. However, these factors are not often analyzed explicitly and simultaneously.^59^, ^60^, ^61^, ^62^ Our work addressed this gap by proposing an integrated, direct analysis of all 3 variables. To our knowledge, ours is the first study to include them jointly within this specific analytical framework. We chose an unsupervised approach to avoid overfitting, which is particularly relevant in supervised models with small sample sizes, while allowing the exploration of data structure without imposing predefined classifications.

Through 3-D analysis, we deciphered the complex interactions between patient age, specific IgE levels, and wheal sizes, providing a detailed overview that could permit new insights into allergic responses and the effectiveness of immunotherapy treatment. Our primary goal was to study the relationships between the considered variables and observe any changes in these relationships after immunotherapy treatment. To do this, we applied *k*-means clustering to divide the sample into two distinct clusters—a parameter we specifically configured.

The concept of clusters provides an effective method for analyzing the physiologic correlation between age, specific IgE levels, and the average wheal surface area, allowing for a detailed understanding of patient values in comparison to the general population of Apulia (Italy), with particular focus on the province of Bari. The ability to compare clusters before and after immunotherapy permits analysis of how treatment influences the considered variables. It is possible to identify significant changes in our patient group, thus allowing us to assess the effectiveness of the treatment and to identify potential predictors of a good response.

The effect of immunotherapy on the allergic response in the patient pool can be analyzed through 3 modes of 3-D clustering, as follows.

#### Original data

In Fig 4, *A* and *B,* related to the preimmunotherapy phase, the clustering algorithm identified two distinct groups in the dataset. The colors (purple and blue) represent the different clusters formed by the *k*-means algorithm; each point is colored according to the label of the cluster to which it belongs.

In the preimmunotherapy phase (Fig 4, *A*), the colors of the points in the clusters highlight two distinct patient groups: purple identifies those with low specific IgE levels and small wheal size, regardless of age, while blue represents patients with high specific IgE levels and large wheal size, also without age-related differences.

It is evident that points with higher IgE levels have larger wheal sizes, indicating a stronger allergic response. Typically, especially in allergic patients, there is a positive correlation between specific IgE levels and wheal size, as a higher level of specific IgE indicates a stronger immune response to the allergen, which could lead to a larger wheal. After immunotherapy (Fig 4, *B*), on average, the wheal size decreases, indicating a reduction in allergic reactivity. Consequently, specific IgE levels should also decrease, signaling reduced sensitivity to allergens. The age variable shows that allergic responses can vary among different age groups. The clusters may reveal patterns such as younger individuals clustering separately from older individuals according to their immune response. Age can influence the immune response: Younger individuals may have a more reactive immune system compared to older individuals, which is reflected in both specific IgE levels and wheal size. In the postimmunotherapy phase, the purple cluster identifies patients who showed a significant reduction in wheal size and specific IgE levels, indicating a good response to treatment. In contrast, the blue cluster represents patients with a more limited reduction, where both parameters remain at medium-high values.

When we compared the post data with the pre data, changes in the allergic response after the intervention could be identified. A significant reduction in specific IgE levels or wheal size in the post compared to pre data indicates the effectiveness of the intervention. Clustering analysis by *k*-means allows identifying groups of individuals with similar responses to immunotherapy: individuals with a significant reduction in wheal size and specific IgE levels after immunotherapy, indicating a good response to treatment, and individuals with a smaller or no significant reduction in wheal size and specific IgE levels, suggesting a less effective response to immunotherapy. Therefore, analyzing the interactions between variables in the post data compared to the pre data allows for evaluating the effectiveness of immunotherapy, with a decrease in wheal size and specific IgE levels indicating a positive response to the treatment, influenced by the age of the individuals.

#### Normalized data with different weights

A 3-D representation of the data related to the patient pool was generated, and the *k*-means clustering algorithm was applied with two clusters, both before and after immunotherapy treatment.

Before clustering, the data underwent two-step preprocessing. First, standardization was performed (transforming each variable to have a mean of 0 and a variance of 1) to ensure that all 3 variables (specific IgE levels, allergen-induced wheal surface area, and patient age) contributed equally regardless of their original measurement scales. Next, a normalization step with differentiated weights was applied, assigning a different importance to the variables on the basis of the evidence reported in scientific literature. Specifically, greater importance was attributed to the surface of the wheal caused by the allergen (with a weight of ⅔), with an intermediate weight to specific IgE (⅓), while age was maintained at its real weight. In this way, it was possible to clearly see the distribution of the variables in the various groups and understand how the 3 variables interacted with each other. This normalization allowed us to emphasize the most significant variables for the allergic response, ensuring that the clustering more accurately reflected the relative contribution of each parameter to patient stratification. The analysis, conducted before and after immunotherapy, allowed to observe any variations in the distribution of the patients between the two identified groups, providing more precise information on the effects of the treatment.

The results obtained by applying *k*-means clustering with two clusters to the normalized data showed a distribution that is almost superimposable to that obtained using the original data (Fig 4, *C* and *D*). This suggests that despite the different scale of importance assigned to the variables through the normalization made on the basis of the evidence in the literature, the division of patients into the two groups remained substantially unchanged. This result indicates that the intrinsic relationships between the analyzed variables are already well delineated in the original data and do not undergo significant changes in terms of clustering after the reassignment of the weights.

In the 3-D representation (Fig 4, *A-D*), the separation of clusters occurs mainly along the IgE and wheal axes, with no appreciable gradients along the age axis. This spatial configuration provides a qualitative confirmation of the quantitative evidence reported in Fig 3, *B-H,* reinforcing the observation that age exerts a negligible influence compared to the other two variables. This aspect strengthens the robustness of the model in identifying distinct groups of patients, regardless of the normalization methodology adopted. Moreover, Fig 4, *C* and *D,* shows that the greater homogeneity observed between clusters after immunotherapy (60.61% vs 39.39%, compared to the previous 81.82% vs 18.18%) suggests a modulatory effect of the treatment on the immune response, reducing interindividual variability.

Clustering revealed two distinct clinical profiles, objectively identifying patients with good versus partial response to immunotherapy. This stratification supports the use of AI to optimize follow-up and personalize treatment according to individual response. Importantly, the partial overlap of clusters at the two time points did not indicate a lack of treatment efficacy but rather suggested that immunotherapy acts by gradually modulating clinical parameters, without necessarily causing abrupt transitions between subgroups. This progressive shift reflects the complex, dynamic nature of immune adaptation and highlights the potential of clustering as a tool for longitudinal monitoring and early identification of patients who may benefit from intensified therapeutic strategies.

### Treatment effectiveness

This study contributes to a better understanding of the dynamics underlying allergic skin reactions and the effectiveness of immunotherapy in their treatment. When comparing Fig 1, *A,* and Fig 1, *B,* it is observed that patients predominantly distribute in an area characterized by lower levels of specific IgE and a reduced wheal surface. Therefore, immunotherapy has the effect of homogenizing the patients’ immune response, achieving the set goal of reducing individual variability in allergic responses. This result is highly important.

As widely documented in scientific literature, patients with a high susceptibility to severe allergic reactions to Hymenoptera venom can experience a significant reduction in their allergic reactivity after receiving immunotherapy treatment.^63^^,^^64^ Consequently, the effectiveness of the treatment and the immune response will be observed in both young individuals and adults. Additionally, the variation in specific IgE levels before and after immunotherapy has been studied.

It was found that using clustering to evaluate the effectiveness of immunotherapy treatment provides a deeper insight compared to traditional statistical analyses, allowing the identification of homogeneous groups of patients with different clinical responses. This approach enables patient stratification on the basis of changes in clinical parameters before and after treatment, facilitating the understanding of response dynamics and the definition of subgroups characterized by varying sensitivities to therapy. Specifically, clustering distinguishes patients who experience effective response to treatment (Fig 4, *B, purple*) from those experiencing partial or suboptimal responses (Fig 4, *B, blue*), thus providing useful information for clinical monitoring and potential subsequent therapeutic decisions. Early identification of patients with a poor response to treatment offers the opportunity to promptly intervene with therapeutic adjustments, such as intensifying immunotherapy or introducing complementary therapies.

Cluster analysis can also reveal hidden information about baseline characteristics that influence treatment response, such as differences in immunologic profiles or genetic variations that may be associated with varying drug sensitivity. This paves the way for future studies aimed at further personalizing the therapeutic approach according to each patient’s profile, thereby improving the overall effectiveness of treatment and minimizing the occurrence of side effects. The results of the quantitative analysis via the Müller scale indicate a significant clinical improvement in patients undergoing immunotherapy for Hymenoptera venom. Before treatment (Fig 1, *C*), the majority of patients exhibited severe reactions (scores of 3 and 4), highlighting a high sensitivity to the venom. No patient was classified with a score of 1, suggesting that all participants experienced a certain severity in allergic reactions. After immunotherapy (Fig 1, *D*), the complete absence of patients with scores of 3 and 4 and the prevalence of scores of 1 indicate a marked reduction in severe allergic reactions. Only one patient maintained a score of 2, representing a moderate but still significant improvement compared to baseline. These data strongly support the efficacy of immunotherapy in reducing the severity of allergic reactions to Hymenoptera venom, suggesting that this treatment can lead most patients to a state of tolerance or clinically minimal response.

This project represents a pioneering advancement in the field of allergenic immunotherapy because it involves the use of an AI-based methodology.

### Limitations

Some methodologic considerations must be kept in mind. The sample is focused but numerically limited (30 patients and 33 observations), derives from a single center, and includes patients with response only; this may reduce estimate precision and limit the generalizability to other populations. The temporal heterogeneity between pre and post assessments typical of real-world practice, together with follow-up variability, may have introduced modest sources of variability.

Analytically, clustering was set to *k* = 2 to maximize clinical interpretability and used weights informed by the literature and our experience (wheal ⅔, IgE ⅓, age at its natural weight). Sensitivity analyses (eg, varying *k,* alternative algorithms, stability/bootstrapping) and external validation were not within the exploratory scope of this study, and are planned as future developments.

Looking ahead, larger, multicenter cohorts with patients from diverse geographic/bioclimatic areas could strengthen generalizability, improve model calibration and accuracy, and enable assessment of environmental heterogeneity (eg, exposure to different Hymenoptera species, sting patterns, demographic factors), thereby reducing potential center-specific bias. Overall, these elements do not undermine the internal consistency of the findings or the exploratory conclusions; rather, they define their application scope and motivate further investigation.

### Future developments

The results of this study not only enhance our understanding of allergic skin reactions but also may significantly influence future clinical guidelines and therapeutic strategies, permitting treatment to be tailored to patients’ individual characteristics.

Observing the effectiveness of immunotherapy treatment in patients with high susceptibility to allergic reactions to Hymenoptera venom highlights the importance of this treatment modality in managing skin allergies. The clustering division shows that before treatment, patients are differentiated by specific IgE levels and skin reactivity. After immunotherapy, the purple cluster represents patients who experienced a significant reduction in these parameters, indicating a good response to treatment, while the blue cluster includes those who showed a more limited reduction, with values still in the medium-high range (Fig 4, *B*). This differentiation supports the idea that immunotherapy does not produce uniform effects in all patients and suggests the importance of identifying subgroups of patients with different responses via clustering analysis in order to further personalize the therapeutic approach.

The use of clustering not only supports a more accurate assessment of the effectiveness of immunotherapy but also represents a key tool for developing personalized treatment strategies, optimizing the allocation of clinical resources, and improving patient outcomes.

**Clinical implication**

ML combined with objective wheal measurement enhances the evaluation of venom immunotherapy effectiveness, enabling personalized patient stratification and improving allergy management strategies.

## Disclosure statement

Declaration of generative AI and AI-assisted technologies in the writing process: ChatGPT 4.0 (OpenAI) was used for linguistic reasons to translate terms from Italian to English. No content was generated by AI.

Disclosure of potential conflict of interest: The authors declare that they have no relevant conflicts of interest.

## Figures and Tables

**Supplementary Fig E1:**