# Supplementary material for: Response to venom immunotherapy: Exploratory retrospective machine learning clustering analysis
Source: J Allergy Clin Immunol Glob. 2026 Jan 29;5(3):100651. doi: 10.1016/j.jacig.2026.100651 (PMC12950403; doi:10.1016/j.jacig.2026.100651)
Supplement: Figure legend [file mmc1.docx]

**SUPPLEMENTAL FIGURE LEGENDS:**

**Supplemental Figure 1:**

Visualization of observational units classified according to Müller grades, recorded before and after immunotherapy. Pre-treatment observations correspond to grades II (**A**), III (**B**), and IV (**C**), while post-treatment observations correspond to grades I (**D**) and II (**E**). For each grade, individual observations were plotted in a three-dimensional space defined by wheal surface area, specific IgE levels, and age.
